# Comment on “The Role of Medications in Causing Dry Eye”

**DOI:** 10.1155/2018/7396982

**Published:** 2018-09-30

**Authors:** Nardine Nakhla, Rosemary Killeen

**Affiliations:** School of Pharmacy, University of Waterloo, 200 University Ave W., Waterloo, ON, Canada N2L 3G1

We came upon the article titled “The Role of Medications in Causing Dry Eye” [1], during a recent literature search conducted for a continuing education program we recently produced on the management of dry eye disease.

In reviewing this article, we noted a number of errors, particularly in Tables 3 and 4. Specifically, many of the drugs are misclassified and/or allocated to incorrect therapeutic drug classes, and accordingly, corrigendum has been published.

Please also note the following errors:

- Anti-arrhythmic was misspelled in the publication (“antiarrythmic”)
- Hyoscine methobromide was listed as an alternate name for methscopolamine, but these two agents are not equivalent
- Chlorthalidone was misspelled in the publication (“chlortalidone”)

Please also note that we did not review the literature behind the drugs listed under “drugs secreted in tears” and are therefore unable to validate these claims.

While we do not question the likelihood that these medications may cause or aggravate dry eye, the fact that they are not appropriately classified or identified may lead to unintentional errors or misconceptions by practitioners, students, or patients not familiar with these medications.

As this article has been cited by a number of other papers in the relevant literature, we appreciate your attention to the content updates provided.

Our project focuses on interprofessional collaboration between pharmacists and eye care professionals, and in that spirit, we hope that this information is of value to you.

The list of our findings is presented in Table 3.

The list of our findings is presented in Table 4.

## Figures and Tables

**Table 3:** Systemic drugs probably causing or aggravating dry eyes.

Drug name	Drug class listed in publication	Actual drug class
Acebutolol	Beta-agonist	Beta-blocker with intrinsic sympathomimetic activity
Alfuzosin	Antiandrogen	Alpha_1_-blocker
Atenolol	Alpha-agonist	Beta-blocker
Atropine	Anti-arrhythmic	Anticholinergic; ophthalmic agent, mydriatic
Brompheniramine	Antipsychotic	Histamine H_1_-antagonist, first generation
Chlorpheniramine	Antipsychotic	Histamine H_1_-antagonist, first generation
Chlorthalidone	Thiazide	Diuretic, thiazide-related
Clemastine	Antipsychotic	Histamine H_1_-antagonist, first generation
Clofazimine	Antileprosy	Antibiotic
Clonidine	Beta-blocker	Alpha_2_-adrenergic agonist
Cyproheptadine	Antipsychotic	Histamine H_1_-antagonist, first generation
Dexchlorpheniramine	Antipsychotic	Histamine H_1_-antagonist, first generation
Diphenhydramine	Bronchodilator	Histamine H_1_-antagonist, first generation
Doxazosin	Antiandrogen	Alpha_1_-blocker
Doxylamine	Antispasmodic/antimuscarinic	Histamine H_1_-antagonist, first generation; ethanolamine derivative
Dronabinol	Cannabinoids	Antiemetic; appetite stimulant
Homatropine	Anti-arrhythmic	Anticholinergic; ophthalmic agent, mydriatic
Hyoscine (scopolamine)	Anti-arrhythmic	Anticholinergic; ophthalmic agent, mydriatic
Hyoscine methobromide	Anti-arrhythmic	Antimuscarinic/antispasmodic
Indapamide	Thiazide	Diuretic, thiazide-related
Ipratropium	Anti-arrhythmic	Anticholinergic
Metolazone	Thiazide	Diuretic, thiazide-related
Prazosin	Anti-arrhythmic; beta-blocker	Alpha_1_-blocker
Primidone	Sedative and hypnotic	Anticonvulsant; barbiturate
Promethazine	Antipsychotic	Histamine H_1_-antagonist, first generation
Tamsulosin	Antiandrogen	Alpha_1_-blocker
Terazosin	Antiandrogen	Alpha_1_-blocker
Tolterodine	Anti-arrhythmic	Anticholinergic

**Table 4:** Topical ocular drugs that may cause or aggravate dry eye.

Drug name	Drug class listed in publication	Actual drug class
Apraclonidine	Adrenergic agonist	Alpha_2_-agonist
Brimonidine (ophthalmic)	Adrenergic agonist	Alpha_2_-agonist; antiglaucoma
Dapiprazole	Miotic	Alpha-blocker
Dipivefrine	Prostaglandin	Prodrug of epinephrine
Naphazoline (ophthalmic)	Decongestant	Alpha_1_-agonist; vasoconstrictor; imidazoline derivative
Tetryzoline (ophthalmic)	Decongestant	Adrenergic agonist; vasoconstrictor; imidazoline derivative
